# Health Belief Model constructs and teachers’ use of food rewards

**DOI:** 10.1017/S1368980025101407

**Published:** 2025-10-27

**Authors:** Elizabeth Daniels, Janelle Elmore, Kelly Whitehair, Kevin Sauer, Jennifer Hanson

**Affiliations:** 1 School of Health Sciences, Kansas State University, Manhattan, KS, USA; 2 Elmore Consulting LLC, Columbia, MO, USA; 3 Department of Nutritional Sciences, University of Oklahoma Health Sciences, Oklahoma City, OK, USA; 4 The School of Biological Sciences, Louisiana Tech University, Ruston, LA, USA

**Keywords:** Nutrition policy, Health Belief Model, Rewards, School teachers, Psychological theory

## Abstract

**Objective:**

Classroom celebrations and food rewards are substantial sources of unhealthy foods within the school environment in the USA. This study was designed to describe classroom food reward practices and examine the association between food rewards and constructs of the Health Belief Model (HBM).

**Design:**

An online survey using summated scales of food reward frequency and HBM constructs.

**Setting:**

The online survey was distributed to elementary schools throughout seven Midwestern states from November through December 2023.

**Subjects:**

Elementary school teachers (*n* 256).

**Results:**

Candy was the most frequently used food reward with the majority of teachers (55·9 %) reporting they utilised candy at least ‘sometimes’. Bi-variant analysis revealed food reward frequency was positively correlated with perceived barriers to refraining from the use of food rewards (*r* = 0·47, *P* < 0·01) and negatively correlated with policy cues to action (*r* = −0·22, *P* < 0·01). Multiple regression analysis predicted food reward frequency (*R* = 0·47, *F* (3247) 23·62, *P* < 0·001), but only perceived barriers (*β* = 0·45; *P* < 0·001) contributed significantly to the prediction.

**Conclusion:**

Classroom food rewards are common, and perceived barriers (but not perceived threat or policy cues) were associated with food rewards among this sample of teachers. Reducing barriers to refraining from the use of food rewards may begin to reduce the practice of using classroom food rewards.

Concerns over childhood weight and food security in the USA have led to an overhaul of the school food environment through federal mandates such as the Healthy Hunger Free Kids Act of 2010^(1)^. The Healthy Hunger Free Kids Act of 2010 and the subsequent Final Rule published in 2016 modified the nutritional standards of foods served to students and required local educational agencies address student well-being through strengthened written local wellness policies^(2)^. In early 2024, the U.S. Department of Agriculture announced additional changes to strengthen nutritional standards in an effort to reduce the sodium and added sugars served throughout the school day^(3)^. While these new guidelines aim to improve the healthfulness of the school food environment, they do not explicitly address all sources of food available to students while at school, specifically foods provided by teachers within the classroom as rewards or incentives. Despite guidance discouraging the use of food rewards^(4)^, children are regularly offered food as a means to encourage academic success or modify classroom behaviour^(5–9)^.

Previous studies have found teacher-held beliefs to be supportive of a healthy school environment^(5,7,8)^. Yet, celebrations and classroom food rewards are substantial sources of unhealthy food^(10)^, and educators often engage in the use of highly palatable foods to reward or motivate their students^(5,7–9)^. While food rewards may produce short-term classroom results, they may also have unintended consequences^(11)^. Highly palatable foods that are used as rewards have been shown to override basic homeostatic energy controls as well as decrease desirability of more healthful alternate foods^(12,13)^. Additionally, this practice may alter food-related habits throughout the lifespan^(14)^. Concerns surrounding the use of food as a reward include decreased intrinsic motivation to achieve tasks, increased risk for disordered eating patterns, as well as eating in the absence of hunger, leading to unintended weight gain^(11,13)^.

Despite policies limiting or banning the use of food rewards^(15)^, a dissonance between wellness policies and school-level practices exists^(16–18)^. Teachers play an important role in the decision about what foods are accessible within their classrooms; therefore, it is important to further investigate the intentions of teachers in relation to this behaviour. While much of the current literature has reported the use and frequency of food as rewards, only one previous study used a theory of health behaviour to better understand teachers’ motivations. In this study, researchers used the Theory of Planned Behaviour to measure behavioural beliefs, normative beliefs and control beliefs and found that all three primary determinants contributed significantly to teachers’ behaviour and the use of food rewards in their classrooms^(19)^.

Gaining information using a health theory approach provides valuable information on how to best design and implement effective interventions. To improve upon the paucity of research on the topic of classroom food rewards and to investigate the attitudes and intentions of teachers using theory, a theoretical framework was developed based upon the Health Belief Model (HBM). The HBM is one of the oldest and most widely used health behaviour theories and has been used both to understand behaviour and inform training and education in public health^(20)^. Originally developed to explain limited participation in screening and prevention services^(21)^, the HBM has been applied to an array of nutrition-related outcomes such as healthy eating^(22)^, enrollment in a diabetes prevention program^(23)^ and safe food-handling behaviours^(24)^.

The HBM is a health theory, which addresses behaviours through six constructs: perceived susceptibility, perceived severity, perceived benefits, perceived barriers, cues to action and self-efficacy^(20)^. According to the theory, healthful behaviours are more likely to occur when individuals see themselves as susceptible to a given condition and perceive the condition as serious, believe the healthful behaviours are of benefit, believe the benefits of the behaviours exceed the costs and believe they have the ability to successfully carry out the behaviour. Cues to action (e.g. warning labels, postcard reminders) are considered activation factors that influence behaviour by prompting action^(21)^.

As described by Hiebert et al.^(25)^, theoretical frameworks should be customised, and while the HBM served as the foundation for the theoretical framework for this study, not all constructs were a good fit for the issue at hand. For example, perceived susceptibility, which refers to how individuals perceive their personal risk of developing a condition did not align well with the consequences of providing food reward, which would be conditions experienced by students but not by teachers.

Therefore, we selected three constructs (i.e. perceived barriers, perceived threat and cues to action) that were both a good theoretical fit and suitable for intervention. Perceived barriers have been shown to consistently perform as strong predictors of health behaviours^(26)^, and interventions targeted at reducing barriers are fundamental to public health nutrition^(27,28)^. Perceived threat measures have also been shown to be associated with a variety of health-related outcomes^(22,24,29)^. However, little is known of how teachers perceive the consequences of providing classroom food rewards, making an improved understanding of this concept an important first step in designing effective interventions. Lastly, while cues to action can exist in many forms, this broad construct has been associated with a variety of health-related outcomes^(23,24)^. Public policy, an essential component of public health,^(30)^ includes school nutrition-related policies with the potential to influence both the school food environment as well as eating behaviours.

Published details regarding food reward practices (e.g. food types and occasions) are limited, and despite the HBM’s wide usage in research, the current literature is void of studies in which this model has been applied to the practice of using food rewards in elementary school classrooms. As such, the purpose of this study was to describe classroom food reward practices (i.e. food types and frequencies) and measure the association between three select HBM constructs (i.e. perceived threat, perceived barriers and cues to action) and the use of food rewards within elementary school. It was hypothesised that the use of classroom food rewards would be (a) negatively associated with the perceived health threat of classroom food rewards, (b) positively associated with the perceived barriers to refraining from the use of food rewards and (c) negatively associated with policy cues to action restricting the use of classroom food rewards.

## Methods

### Study design and participants

This was a cross-sectional survey of a convenience sample of elementary school teachers located in seven Midwestern states including Iowa, Kansas, Minnesota, Missouri, Nebraska, North Dakota and South Dakota. Elementary school was defined as kindergarten through fifth grade. Inclusion criteria included survey respondents indicating they (a) were over the age of 18, (b) licensed as a teacher in one of the seven states and (c) had classroom instruction for grades kindergarten through fifth as their primary professional responsibility. Excluded from the study were principals, administrators, paraeducators and specialised instructors (e.g. music, physical education and art teachers).

### Recruitment strategies

State government websites for each state included in this study were extensively searched for educator contact information. An email message stating the purpose for the study and containing the survey link was sent to publicly available email addresses for public school teachers, principals and school administrators in all seven selected states. Within the email was an invitation to take the survey or, in the case of principals and administrators, a prompt to forward the study details to teachers working within their buildings or districts. A follow-up message was sent 2 weeks after the initial email invitation. The opening page of the survey stated participation was completely voluntary and that individuals could remove themselves from the survey at any time. Consequently, informed consent was implied. Incentives were provided in the form of a lottery system with eighteen $100·00 gift cards available for those who completed the survey. A prompt to provide contact information to be entered to win a gift card was required. Survey responses were collected during November and December of 2023. The study protocol was reviewed and deemed exempt by the Kansas State University Committee on Research Involving Human Subjects/Institutional Review Board.

### Survey instrumentation

The survey was developed to (a) screen participants for eligibility, (b) collect participant demographic information, (c) describe current classroom food reward practices and (d) measure the relationship between perceived threats, perceived barriers and cues to action and the frequency of food reward practices within elementary school. Before dissemination, the survey received expert feedback from nutrition researchers (*n* 2) and professional educators (*n* 3) who were asked to review the survey for readability, comprehensibility and relevance. The survey was then edited to reflect expert feedback and subsequently pilot-tested among fifty-three teachers working within the state of Kansas. Adjustments were made after pilot-testing to clarify eligibility, to eliminate redundancy and to elaborate upon the perceived barriers items.

The final survey contained forty-one survey items including three screening questions, one attention check question and two questions related to participants’ optional enrollment into the incentive drawing. The survey, which was designed to be accessed online and self-administered, was disseminated in Qualtrics (Version November 2023, Provo, UT). Completion of the survey took approximately 12 min.

### Measurement instruments

#### Food reward types

A series of six items was created to portray the food reward types used within the participants’ classrooms. Specifically, teachers were asked how often they offered each of the following as a reward within the last 30 d: ‘candy’, ‘chips/pretzels/crackers’, ‘gummy (fruit) snacks’, ‘soda/pop/juice’, ‘granola/snack bars’ and ‘other’ foods. Frequency scale response categories were ‘never’, ‘rarely’, ‘sometimes’, ‘often’ and ‘daily’.

#### Food rewards use

A set of five items was designed to measure frequency of use of classroom food rewards. In this set of survey items, teachers were asked to indicate how often they provided food rewards for a series of different scenarios including good behaviour, attendance, task completion, milestones and doing well on assignments. Frequency scale response categories were ‘never’, ‘1–2 times a month’, ‘1–2 times a week’, ‘3–4 times a week’ and ‘daily.’ Responses were assigned a value from 1 (‘never’) to 5 (‘daily’), with assigned values increasing as frequency increased. Principal component analysis resulted in the detection of a single dimension and the retention of all five items. Cronbach’s alpha for the resulting Food Rewards Use Scale was 0·72.

#### Perceived threat of classroom food rewards

A set of six items was developed to measure the perceived health threat associated with the use of classroom food rewards. The set of items included both positive (e.g. ‘classroom food rewards will distort a child’s relationship with food’) and negative (e.g. ‘classroom food rewards are harmless’) statements. A five-point Likert scale was used to measure participant’s level of agreement with each statement with responses ranging from ‘strongly disagree’ to ‘strongly agree’. Negative statements were re-coded so that higher scores indicated greater perceived threat. Principal component analysis led to the detection of one dimension with the retention of all six items. Cronbach’s alpha for the resulting Perceived Threat Scale was 0·86.

#### Perceived barriers to refraining from the use of classroom food rewards

A set of nine items was created to measure perceived barriers to refraining from the use of classroom food rewards. Participants’ level of agreement was measured using a five-point Likert scale with responses ranging from ‘strongly disagree’ to ‘strongly agree’. Responses were assigned values increasing as perceived barriers increased. Principal component analysis of the items resulted in the identification of two dimensions. The first dimension resulted in the six item Perceived Barriers Scale with a Cronbach alpha of 0·77. The second dimension consisted of three items related to organisational support for avoiding classroom food rewards (i.e. ‘I do not have administrator support’, ‘I do not have the resources to afford other rewards for my classroom’ and ‘I have never been provided training on non-food rewards’). Because the Cronbach alpha for these items was 0·42, the second dimension was not used to operationalise perceived barriers.

#### Policy cues to action

A two-item set of statements was created to measure exposure to policy cues prompting refrainment from the use of classroom food rewards. Teachers were asked to indicate their level of agreement on a five-point Likert scale. Statements included ‘my school district has a policy stating food should not be used as a reward,’ and ‘my school has a policy stating food should not be used as a reward.’ Principal component analysis of the cues to action items resulted in the identification of single dimension with retention of both items. Although the Cronbach alpha level for the Cues to Action Scale was found to be high (*α* = 0·97), the statements were determined to test different concepts.

### Data analysis

Descriptive analyses were performed to characterise the sample, portray the reported use of specific food reward types and summarise responses to each set of questions designed to operationalise food reward use as well as the three HBM constructs under study. For each question set, principal component analysis with varimax rotation and Cronbach’s alpha values were determined to assess construct validity and internal consistency, respectively. Summary scores for each of the scales were created by totaling individual item response scores. Demographic and other teacher characteristic responses were collapsed into meaningful categories (e.g. years of teaching experience ≤ 10 years *v*. > 10 years) to allow for comparison of food reward behaviours using independent sample *t* tests. Relationships between the resulting scales scores were explored using Pearson correlation coefficients. Multiple regression analysis with stepwise elimination was conducted with perceived threat, perceived barriers and cues to action scale scores as independent variables and food reward scale scores as the dependent variable. The elimination of variables from the regression analysis was based on a significance level of *P* < 0·10. For all other analysis, the significance level was set at *P* ≤ 0·05. All data were analysed using IBM SPSS Statistics for Windows, Version 29.0 (IBM).

## Results

Four hundred thirty-nine individuals accessed the survey of which 280 indicated they met the study inclusion criteria. Of those that met the inclusion criteria, only individuals who completed one or more scale questions and correctly answered the attention check question (i.e. ‘2 + 2 =’) were included in the study. In all, 256 teachers representing seven states and ninety-two different school districts were included. Most identified themselves as being white (92·6 %) and female (91·4 %). There was relatively even representation among all grade levels. However, 20·7 % indicated that they taught a grade other than kindergarten through fifth. The ‘other’ category was included in the list of choices to accommodate teachers of rural areas who may teach multiple grades within the same classroom. Most teachers had either a bachelor’s (48·8 %) or a master’s degree (47·3 %), and just over one-half (51·6 %) had been teaching for 10 years or less. (Table 1).

**Table 1:** Characteristics of elementary school teachers survey sample (*n* 256)

Variable		*n*	%
Gender			
	Male	20	7·8
	Female	234	91·4
	Prefer not to answer	2	0·8
Ethnicity			
	White	237	92·6
	Non-white	11	4·4
	Prefer not to answer	8	3·1
Grade taught			
	Kindergarten	43	16·8
	1st grade	24	9·4
	2nd grade	31	12·1
	3rd grade	37	14·5
	4th grade	33	12·9
	5th grade	35	13·7
	Other	53	20·7
Student to teacher ratio			
	≤ 1:10	45	17·6
	1:11–1:15	37	14·5
	1:16–1:20	81	31·6
	1:21–1:25	78	30·5
	≥ 1:26	15	5·9
Teaching experience			
	≤ 1 year	10	3·9
	2–5 years	69	27·0
	6–10 years	53	20·7
	11–16 years	42	16·4
	17–20 years	15	5·9
	21–25 years	32	12·5
	≥ 26 years	35	13·7
Education			
	Bachelors	125	48·8
	Masters	121	47·3
	Professional	8	3·1
	Doctorate	2	0·8
State			
	Iowa	20	7·8
	Kansas	32	12·5
	Minesota	7	2·7
	Missouri	61	23·8
	Nebraska	5	2·0
	North Dakota	39	15·2
	South Dakota	24	9·4
	Unknown	68	26·6

### Food reward practices

Only 14 % of respondents indicated that they had not used any form of food reward in the 30 d prior to the survey. Candy was the food most frequently used, with the majority (55·9 %) of teachers reporting they used candy as a reward at least ‘sometimes’ during the previous 30 d. The proportions of teachers that used chips, pretzels or crackers (30·8 %), gummy (fruit) snacks (26·6 %), granola bars (24·2 %) or drinks like soda, pop or juice (6·6 %) at least ‘sometimes’ during the previous 30 d are noted in Table 2. Seventy-three (73·3 %) percent of teachers reported using food to celebrate a milestone such as the last day of school or the completion of an exam or project, while 62·5 % used food as recognition for good behaviour. Only 6·6 % of teachers reported using food as a reward for school attendance. See Table 3.

**Table 2:** Food reward types used by teachers during previous 30 d (*n* 256)

Food reward	Never		Seldom		Sometimes		Often		Daily	
	*n*	%	*n*	%	*n*	%	*n*	%	*n*	%
Candy	49	19·1	64	25·0	88	34·4	41	16·0	14	5·5
Chips/pretzels/crackers	134	52·3	43	16·8	50	19·5	21	8·2	8	3·1
Gummy (fruit) snacks	145	56·6	43	16·8	41	16·0	23	9·0	4	1·6
Granola/snack bar	155	60·5	39	15·2	41	16·0	17	6·6	4	1·6
Soda/pop/juice	191	74·6	48	18·8	15	5·9	1	0·4	1	0·4
Other	167	65·2	37	14·5	36	14·1	11	4·3	5	2·0

**Table 3:** Teachers’ use of classroom food rewards

Food Rewards Use Scale (*α* = 0·72)	Mean score	sd	Never		1–2 times per month		1–2 times per week		3–4 times per week		Daily	
			*n*	%	*n*	%	*n*	%	*n*	%	*n*	%
I offer my students food rewards for good behaviour.	2·02	1·06	96	37·5	93	36·3	42	16·4	15	5·9	10	3·9
I offer my students food rewards for good attendance.	1·09	0·41	239	93·4	13	5·1	1	0·4	3	1·3	0	0
I offer my students food rewards when tasks are completed on time.	1·42	0·90	194	75·8	35	13·7	15	5·9	5	2·0	7	2·7
I offer my students food rewards to celebrate a milestone (e.g. last day of school, completion of an exam, completion of a project).	1·88	0·72	71	27·7	155	60·5	20	7·8	9	3·5	1	0·4
I offer my students food rewards when they do well on an assignment, quiz or test.	1·46	0·78	173	67·6	55	21·5	21	8·2	6	2·3	1	0·4

Mean scores with standard deviations and response frequencies with percentages (*n* 256). Mean scores were calculated by assigning increasing values based on increased frequency of use of classroom food reward (Daily = 5; 3–4 times per week = 4; 1–2 times per week = 3; 1–2 times per month = 2; Never = 1).

Food Rewards Use Scale scores did not differ significantly based on gender (male *v*. female, *t*(20·05) = 0·96, *P* = 0·35); ethnicity (white *v*. all others, *t*(10·26) = –0·50, *P* = 0·63); grade taught (kindergarten through second grade *v*. third grade through fifth grade, *t*(201) = –1·63, *P* = 0·10); teacher to student ratio (≤ 1:20 *v*. > 1:21, *t*(254) = 0·21, *P* = 0·83), years of teaching experience (≤ 10 years *v*. > 10 years, *t*(232·77) = –0·68, *P* = 0·50) or education level (bachelor degree *v*. higher, *t*(254) = –0·27, *P* = 0·79).

### Perceived threat of classroom food rewards

Most teachers either ‘somewhat disagreed’ or ‘strongly disagreed’ that using food rewards would lead to poor eating habits (61·3 %) or would distort a child’s relationship with food (55·5 %). Nearly one-half (42·6 %) either ‘somewhat disagreed’ or ‘strongly disagreed’ that classroom food rewards undermine healthy nutrition practices. Forty-three percent (43·0 %) either ‘somewhat agreed’ or ‘strongly agreed’ that food rewards are harmless, while 43·8 % either ‘somewhat agreed’ or ‘strongly agreed’ that food rewards do not place their student’s health at risk or that using rewards are not a big deal, respectively. See Table 4.

**Table 4:** Health Belief Model construct scale responses

	Mean score	sd	Strongly disagree		Somewhat disagree		Neither agree nor disagree		Somewhat agree		Strongly agree		No response	
			*n*	%	*n*	%	*n*	%	*n*	%	*n*	%	*n*	%
**Perceived Threat Scale (*α* = 0·86)**														
Classroom food rewards undermine healthy nutrition practices.	2·73	1·15	45	17·6	64	25·0	74	28·9	60	23·4	13	5·1	–	–
Classroom food rewards are harmless.	2·79	1·14	17	6·6.	58	22·7	71	27·7	74	28·9	36	14·1	–	–
The use of food rewards will lead to poor eating habits.	2·33	1·06	62	24·2	95	37·1	57	22·3	37	14·5	5	2·0	–	–
Using food as a reward does not place my student’s health at risk.	2·79	1·17	21	8·2	53	20·7	70	27·3	74	28·9	38	14·8	–	–
Rewarding students is not that big a deal to me.	2·73	1·17	22	8·6	47	18·4	63	24·6	88	34·4	36	14·1	–	–
Classroom food rewards will distort a child’s relationship with food.	2·41	1·11	64	25·0	78	30·5	67	26·2	40	15·6	7	2·7	–	–
**Perceived Barriers Scale (*α =* 0·77)**														
Food rewards makes coming to school more fun for my students.	3·08	1·16	38	14·8	29	11·3	76	29·7	91	35·5	17	6·6	5	2·0
My students like getting food as a reward in the classroom.	4·17	0·96	10	3·9	4	1·6	26	10·2	104	40·6	107	41·8	5	2·0
I like providing food rewards to my students.	3·18	1·09	27	10·5	28	10·9	92	35·9	82	32·0	22	8·6	5	2·0
The other teachers in my building use food rewards.	3·95	0·86	5	2·0	8	3·1	44	17·2	131	51·2	63	24·6	5	2·0
My students’ parents think it is a good idea to use food rewards in the classroom.	3·18	0·68	8	3·1	6	2·3	179	69·9	49	19·1	9	3·5	5	2·0
Rewarding students with food makes me feel good.	2·82	0·97	34	13·3	31	12·1	144	56·3	31	12·1	11	4·3	5	2·0
**Cues to Action Scale (*α* = 0·97)**														
My school district has a policy stating food should not be used as a reward.	2·02	1·2	128	50	29	11·3	65	25·4	19	7·4	10	3·9	5	2·0
My school has a policy stating food should not be used as a reward.	1·99	1·19	130	50·8	32	12·5	61	23·8	18	7·0	10	3·9	5	2·0

Mean scores with SD and response frequencies with percentages (*n* 256). Mean scores were calculated by assigning increasing values based on level of agreement with each item (Strongly Agree = 5; Somewhat Agree = 4; Neither Agree nor Disagree = 3; Somewhat Disagree = 2 and Strongly Disagree = 1). Negatively worded perceived threat items were recoded so that increasing values indicating greater perceived threat.

### Perceived barriers to refraining from the use of classroom food rewards

The majority of teachers indicated they observe other teachers using food rewards in their building (75·6 %) and that they believed their students like receiving food rewards (82·4 %). Many teachers (40·6 %) responded that they like providing food rewards but felt neutral when asked if providing food as a reward made them feel good. Nearly 70 % of teachers responded as unsure when asked if they believed that their students’ parents found food rewards a good idea or not, and only 42·1 % believed that the use of food rewards made coming to school more fun. See Table 4.

### Policy cues to action

When asked about policies against the use of food rewards, only 11·3 % ‘somewhat agreed’ or ‘strongly agreed’ that their school district had a policy. Similar responses (11·3 %) were recorded in regard to the question as to whether their school had such a policy. See Table 4.

### Health Belief Model

Bivariant analyses revealed that Food Rewards Use Scale scores were positively correlated with Perceived Barriers Scale scores (*r* = 0·47, *P* < 0·001) and negatively correlated with Cues to Action Scale scores (*r* = −0·22, *P* < 0·001). Food Rewards Use Scale scores were not significantly correlated with Perceived Threat Scale scores.

Multiple regression analysis predicted food reward use (*R* = 0·47, *F* (3247) 23·62, *P* < 0·001), but only barriers to refraining from the use of food rewards (*β* = 0·45, *P* < 0·001) contributed significantly to the prediction.

## Discussion

The aim of this study was to describe classroom food reward practices and compare elementary school teachers’ use of classroom food rewards against key constructs of the HBM. Among the participants in this study, the use of food rewards was a common classroom practice, and candy was the most frequently utilised food reward. In addition, more frequent use of food rewards was associated with greater barriers to refraining from food rewards. Of the barriers, perceptions of a student’s acceptance of food rewards and the knowledge of other teachers’ practices were most prominent. The findings of the current study are similar to that of other studies in which one or more, but not all construct of the HBM, were found to be associated with health-related outcomes^(23,24,29)^.

The frequent use of food rewards was unsurprising as this finding is consistent with other recorded classroom practices in which food was used in some way within the classroom^(5–8,17)^ Using candy specifically may be viewed as a classroom management strategy because of its affordability, portability and perceived acceptability by children. While it may be assumed children prefer candy over other types of rewards, they are unlikely to be asked for a preference before being provided with the reward. In one study, children in grades first through fifth preferred social rewards (e.g. praise from teacher) and activity related rewards (e.g. free time, picking games at recess) over anything tangible including foods and small items^(31)^. This topic was explored in middle and high school students where results were more mixed with students indicating high but fairly equal preferences for snacks, free time and positive notes sent home^(32)^. This preference towards snacks in older students could be due in part to conditioning. If children become accustomed to receiving food as a reward for academic success and engaging in desirable behaviour from parents, guardians and teachers, they may be more likely to come to expect such reward even in the higher grades. These students may also be more cognizant of the possible rewards and their availability. If students prefer other types of rewards and using food rewards are not in observation of best practice, additional steps should be taken towards eliminating the practice.

Interestingly, as an alternative to food rewards, token economies have been discussed as a possible solution. In a token economy, students are given stickers, punch cards, tickets or other small tokens that can then be traded for small prizes or activities such as wearing pajamas to school or watching a movie. Token economies focus on the use of extrinsic motivation and have been shown to elicit short-term desired behaviour^(33)^. However, food rewards are frequently used within the token economies with students being able to exchange their tokens for candy or snack foods^(34)^. In this scenario, food is being used to reinforce a desired behaviour or outcome and therefore should be discouraged or alternatives prizes used.

Of note in the current study is the finding that teachers indicated that they did not believe the practice of using food to reward students was harmful. Educators may know that using food within the classroom is discouraged but may not understand the reason why. Alternatively, they may have been exposed to messaging stemming from industry sponsored research such as that suggesting candy consumption decreases the risk of obesity in children^(35)^ and confectionary treats enhance academic performance^(36)^. Although food rewards are discouraged by the American Academy of Pediatrics^(4)^ at this time there is limited research demonstrating harmful long-term health effects of classroom food rewards. Nonetheless, parental use of food rewards is associated with an increased preference for foods high in fat and sugar^(37)^, an increased intake of energy dense snack foods and a decreased intake of fruit among children^(38)^. Emerging evidence suggests food rewards may elicit a unique neural response within the brain^(39)^ and that children with obesity find energy dense foods particularly rewarding^(40)^. Educating teachers on the risks associated with food rewards would be recommended as increasing perception of threat can effect motivation for engaging in healthier behaviours^(41)^.

Although less frequent use of food rewards was associated with greater exposure to policies restricting food reward use in the bivariate analysis, it was not in the multivariate analysis, and very few teachers indicated that their school or their school district had a policy against the use of food rewards. This finding corroborates previous studies which found that many teachers were uncertain if their school had a classroom food policy^(5)^ and that fewer than one in eight school districts had a policy prohibiting the use of food rewards^(42)^. If there is a policy prohibiting the use of classroom food rewards, the policy may not be well known, or the policy may be not be enforced and therefore disregarded as observed by Girona et al.^(18)^. Likewise, seeing others engage in the use of food rewards may be reinforcing of the behaviour^(19)^. Having a policy could decrease the use of food rewards with the use of strong direct language^(43)^. However, it is unlikely for school policies to be written in a way that exceeds the state or federal standard, and if removal of classroom food reward is not mandated, schools will be unlikely to independently adopt a strict, enforceable policy.

This study had several strengths including the regional nature of the data and the inclusion of teachers from ninety-two different school districts in seven states. The survey sample was predominantly female and non-Hispanic white, which reflects the proportion of elementary teachers in the USA who are female (89·7 %) and non-Hispanic white (79·0 %)^(44)^. Additionally, a majority of respondents had 10 years or less of experience in the field, and work experience has been found to be negatively correlated to the use of food rewards^(5)^.

The scales used, while novel, were evaluated by expert feedback, piloted and underwent evaluations of construct validity and reliability before being used for hypothesis testing. To our knowledge, this study is the first of its kind to apply HBM constructs to the issue of classroom food rewards. While contributing to the sparce research in this area, it should be noted that the use of select variables *v*. the full HBM is a limitation.

Additional limitations of this study include the cross-sectional research design, the non-probability sampling technique and self-reported nature of the data. To reduce non-classroom teachers from participating, screening questions were included; however, 20 % of respondents indicated that they taught something other than kindergarten through fifth grade, which could imply that some teachers teach multiple grades, or that some respondents had primary roles outside of direct classroom instruction. While this study included teachers from across the midwestern region of the USA, dissemination of the survey was contingent on principles and administrators forwarding it. This method allowed for a broader dissemination of the survey; however, it precluded our ability to determine the number of recipients who ultimately received and viewed the survey and may have resulted in a non-representative sample.

Lastly, in measuring food reward types, the reward options listed in the survey did not include nutrient dense foods. While we did ask about ‘other’ food rewards, we did not ask participants to list the ‘other’ foods thereby making it impossible to determine the nutritional value of the ‘other’ foods. Historically, studies examining the reinforcing qualities of food have been primarily limited to foods high in fat or high in sugar and fat^(45)^. While the reinforcing value of an unhealthy snack may be higher than that of a healthy snack^(37)^, the conditions under which the food reward is earned as well as the weight status of the child appear to influence its relative reinforcing value^(40,46)^. Consequently, these points should be taken into consideration when interpreting the current study’s findings.

### Conclusion

Eating habits formed in childhood can last a lifetime, and the school food environment plays an important role in the health and well-being of children^(47–49)^. Adherence to best food practices within the classroom is an important consideration given the amount of time children spend at school. Teachers are in a position to support a healthy school environment and often have authority over the foods accessible while in the classroom. Despite expert guidance to prohibit or discourage the use of food rewards in elementary school classrooms, this practice continues, highlighting the difficulty in its cessation. As discovered in this study, teachers had limited exposure to policies restricting classroom food rewards. This may be due to the lack of policy or may be due to a lack of awareness of such policies. The study findings also suggest that addressing barriers to refraining from the use of food rewards may be a good place to start. However, a more holistic approach including multiple stakeholders (i.e. parents, teachers and administrators), training, and strongly worded policy will likely be required to see an end to this practice.
